# Integrated Transcriptomic and Metabolomic Analysis Deciphers the Molecular and Metabolic Mechanisms Underlying Growth Rate Divergence in Dezhou Donkeys

**DOI:** 10.3390/ani16081271

**Published:** 2026-04-21

**Authors:** Xinhao Zhang, Haijing Li, Xiangnan Zhou, Xianggang Cao, Manna Dou, Changfa Wang, Wenqiang Li

**Affiliations:** 1 Liaocheng Research Institute of Donkey High-Efficiency Breeding and Ecological Feeding, Liaocheng University, Liaocheng 522000, China; zhangxinhao@lcu.edu.cn (X.Z.); 17861820204@163.com (X.Z.); 2510190501@stu.lcu.edu.cn (X.C.); doumanna330@163.com (M.D.); 2 Dong’e Ejiao Co., Ltd., Liaocheng 252200, China; lihaijing@dongeejiao.com

**Keywords:** Dezhou donkey, transcriptomics, metabolomics, multi-omics integration, growth regulatory mechanism

## Abstract

Dezhou donkey is a premium indigenous Chinese livestock breed with high economic value for meat, hide and medicinal uses, and growth rate is a core trait determining farming profitability. However, the molecular and metabolic mechanisms underlying divergent growth rates in this breed have not been fully characterized, with no integrated transcriptomic and metabolomic studies reported. Here, 12 age-matched healthy male Dezhou donkeys were assigned to faster-growing (*n* = 6) and slower-growing (*n* = 6) groups by average daily gain, followed by plasma transcriptome sequencing and untargeted LC-MS/MS metabolomics. We identified 480 differentially expressed genes, with the slower-growing group enriching in immune/inflammatory/apoptotic pathways, and the faster-growing group in energy metabolism and transmembrane transport. Lipids and lipid-like molecules represented the largest proportion (44.9%) of the differential metabolites; the slower-growing group was enriched in lipid peroxidation and pro-inflammatory mediators, while the faster-growing group was enriched in unsaturated fatty acids and antioxidants. Integrated analysis revealed core pathways (cAMP signaling, arachidonic acid/unsaturated fatty acid biosynthesis) and key candidate genes/metabolites. Our findings clarify that excessive lipid peroxidation and inflammatory imbalance restrict growth, while efficient energy metabolism promotes faster growth, providing theoretical support for genetic improvement and precision nutrition of Dezhou donkeys.

## Simple Summary

Dezhou donkeys are important local livestock in China with high economic value, as they are widely used for meat, donkey-hide gelatin and medicinal products. Their growth speed directly affects the breeding benefit, but the internal reasons for the large difference in growth rate are still unclear. In this study, we selected 12 healthy young Dezhou donkeys with the same age and divided them into fast-growing and slow-growing groups. By analyzing their blood samples, we found obvious differences in gene expression and metabolite levels between the two groups. Slow-growing donkeys showed stronger lipid oxidation and higher inflammatory responses, while fast-growing individuals had more efficient energy metabolism and higher levels of beneficial unsaturated fatty acids and antioxidants. The combined analysis further identified key genes and metabolic pathways related to growth. These results explain the molecular mechanism of growth differences in Dezhou donkeys and provide a scientific basis for their better breeding and precise feeding management.

## 1. Introduction

The donkey *Equus asinus* is among the earliest domesticated livestock species and is widely believed to have originated from the domestication and selective breeding of African wild asses *E. asinus africanus* and *E. asinus somaliensis* [1,2]. Throughout history, donkeys have served as important draft and pack animals, driving socioeconomic and cultural development [3], and they have also demonstrated notable commercial value and substantial economic potential in the industrial production of meat, hides, and milk [4]. Compared with beef and mutton, donkey meat offers nutritional advantages including higher levels of crude protein, essential amino acids [5], and unsaturated fatty acids, together with lower total fat and cholesterol, making it a prominent research focus in the development of high-quality livestock meat resources [6,7]. However, industrial mechanization and a lack of standardized breeding systems have constrained the transition to intensive production [8]. Growth rate is a core economic trait in donkey farming that directly determines production cycles and profitability, yet the molecular regulatory differences among individuals with divergent growth performance remain unclear. Elucidating the intrinsic mechanisms underlying growth rate variation through blood-based transcriptomics and metabolomics would not only provide critical theoretical support for germplasm conservation and precision genetic improvement, but also carry practical significance for overcoming industry constraints and advancing the sustainable development of donkey husbandry.

Blood, as the central medium for material transport and signal transduction in the body, can dynamically reflect the physiological and metabolic homeostasis of organs and tissues and, through subtle changes in its components, intuitively reveal differences in individual growth performance [9,10]. It is therefore an ideal biological material for investigating growth regulation in livestock and poultry [11]. Within molecular biology, transcriptomics, owing to its precise characterization of gene expression profiles, has become a key approach for dissecting the mechanisms that govern biological traits [12]. As the principal conduit for nutrient transport and cellular signaling, blood transcriptomic profiles can be effectively linked to the overall physiological state of the organism [13]. Transcriptomic studies in donkeys have shown that this approach can clearly capture dynamic changes in gene expression and has successfully identified functional genes involved in muscle development, skin formation, maintenance of testicular function, lactation, and oocyte maturation, thereby providing important references for molecular breeding in donkeys [4,14]. Meanwhile, blood metabolomics, through systematic monitoring and analysis of the composition and abundance of metabolites in blood, enables in-depth exploration of species-specific characteristics, growth and developmental trajectories, and stress-related metabolic pathways, and facilitates the identification of key metabolites that shape phenotypes [15,16]. Although research on donkey germplasm characteristics and product quality has made progress, comprehensive analyses of blood transcriptomic and metabolomic features in donkeys with differing growth rates and investigations into the molecular mechanisms by which these jointly regulate growth phenotypes remain scarce. This knowledge gap precludes the identification of molecular targets and the establishment of a robust theoretical foundation for the precision improvement of growth performance in donkeys.

The Dezhou donkey is an excellent indigenous Chinese breed with a stable genetic background, superior production traits, and a large population size, providing a solid foundation for genetic improvement and related research [17,18]. In this context, the present study focuses on Dezhou donkeys with different growth rates and conducts comparative analyses of their blood transcriptomic and metabolomic profiles. The aim is to elucidate the molecular mechanisms that regulate differences in growth rate in Dezhou donkeys, thereby providing theoretical underpinnings and technical support for the precise enhancement of growth performance and for promoting the efficient development of the donkey farming industry.

## 2. Materials and Methods

### 2.1. Animals and Sample Collection

Healthy male Dezhou donkeys approximately 9 months of age and of closely matched day-age were selected from a donkey farm in Liaocheng, Shandong, China. The initial average body weight of the 9-month-old donkeys was 141 kg. Animals were individually housed in standardized pens equipped with roofing and fencing, under uniform feeding and management conditions. The donkeys were fed a standardized commercial fattening diet formulated for growing Dezhou donkeys, with a guaranteed minimum crude protein content of 15.00% and a digestible energy level of 10.8 MJ/kg to meet their nutritional requirements. All animals had free access to clean drinking water throughout the trial. Throughout the trial, linear body measurements, body weight, and average daily gain (ADG) were recorded. After 6 consecutive months of feeding (when the donkeys reached 15 months of age), individuals with markedly different growth rates were identified and selected (*n* = 6 per group). The final average body weight was 182 kg in the fast-growing group and 158 kg in the slow-growing group. Blood samples were collected via jugular venipuncture for subsequent transcriptomic and metabolomic sequencing analysis. The samples were snap-frozen in liquid nitrogen and then transferred to a −80 °C ultra-low temperature freezer for storage.

### 2.2. Extraction of Total RNA and Transcriptome Sequencing Analysis

Plasma from two groups of samples was selected for analysis: the slower-growing group D (*n* = 6) and the faster-growing group N (*n* = 6). Total RNA was extracted from these plasma samples using TRIzol reagent (Invitrogen, Carlsbad, CA, USA) according to the manufacturer’s instructions. Briefly, samples were thoroughly homogenized, chloroform was added, and the mixture was centrifuged to separate the phases; the aqueous phase containing RNA was collected, and RNA was precipitated with isopropanol. The RNA pellet was washed with 75% ethanol and finally dissolved in RNase-free water. RNA concentration and purity were assessed using a NanoDrop microvolume spectrophotometer (Thermo Scientific, Waltham, MA, USA). RNA meeting quality criteria was stored at −80 °C for subsequent analyses.

Approximately 3 μg of qualified total RNA was used to construct sequencing libraries with the NEBNext Ultra II RNA Library Prep Kit (New England Biolabs, Ipswich, MA, USA). Briefly, mRNA was purified from total RNA using oligo(dT)-conjugated magnetic beads and then fragmented; first-strand cDNA was synthesized by reverse transcription, followed by second-strand cDNA synthesis. cDNA fragments underwent end repair and 3′ adenylation, after which Illumina paired-end (PE) adapters were ligated. The AMPure XP system (Beckman Coulter, Beverly, MA, USA) was used to select cDNA fragments of 400–500 bp [19]. Adapter-ligated fragments were enriched by PCR with 15 cycles. Final library quality was verified on an Agilent 2100 Bioanalyzer (Agilent Technologies, Santa Clara, CA, USA), and qualified libraries were subjected to paired-end sequencing (2 × 150 bp) on the Illumina NovaSeq 6000 platform.

Transcriptome data were processed as follows: raw sequencing reads (FASTQ format) were filtered with fastp (v0.22.0) to remove low-quality reads, adapter-containing reads, and reads with >5% ambiguous (N) bases. Clean reads were aligned to the reference genome using HISAT2 (v2.1.0). Gene expression levels were quantified with HTSeq (v0.9.1), and read counts were normalized to FPKM (fragments per kilobase of transcript per million mapped reads). Differentially expressed genes (DEGs) were identified with DESeq (v1.38.3) using thresholds of |log2(FoldChange)| > 1 and *p* < 0.05 [20].

Subsequent bioinformatic analyses were performed in R (v4.2.1). Principal component analysis (PCA) was carried out with the vegan package (v2.6.4). Hierarchical clustering heatmaps of gene expression were generated with the pheatmap package (v1.0.12); datasets without biological replicates were transformed using log10(FPKM + 1), whereas datasets with biological replicates were Z-score normalized across samples using Zsample-i = [(FPKM of sample i) − (mean FPKM across all samples)]/(standard deviation of FPKM across all samples). Gene Ontology (GO) enrichment analysis of DEGs was conducted using the g:Profiler online tool. KEGG (Kyoto Encyclopedia of Genes and Genomes) pathway analysis was performed with KOBAS 3.0 and the clusterProfiler package (v4.6.0), with significance defined as *p* < 0.05 [21]. In addition, Gene Set Enrichment Analysis (GSEA, v4.1.0) was used to evaluate enrichment characteristics at the gene-set level.

### 2.3. Metabolite Extraction and LC–MS Detection and Data Analysis

Plasma samples were selected from the slower-growing D group (*n* = 6) and the faster-growing N group (*n* = 6). After slow thawing at 4 °C, 100 μL of plasma was mixed with 600 μL of prechilled methanol–water (4:1, *v*/*v*) containing an internal standard (2-chloro-L-phenylalanine, final concentration 0.06 mg/mL). The mixture was vortexed for 30 s, sonicated in an ice–water bath for 10 min, and then allowed to stand at −20 °C for 30 min. Following centrifugation at 4 °C and 13,000× *g* for 10 min, 200 μL of the supernatant was collected and vacuum-dried. The residue was reconstituted in 150 μL methanol–water (1:4, *v*/*v*), vortexed for 30 s, and sonicated for 3 min. After standing at −20 °C overnight, the samples were centrifuged at 4 °C and 13,000× *g* for 10 min, and 120 μL of the supernatant was collected for LC–MS analysis. In parallel, 10 μL of supernatant from each individual sample in both groups was pooled to prepare a quality control (QC) sample for monitoring extraction stability and reproducibility.

Metabolite separation and detection were performed on a Waters ACQUITY I-Class PLUS UHPLC system (Waters Corporation, Milford, MA, USA) coupled to a Thermo Fisher Q Exactive HF mass spectrometer (Thermo Fisher Scientific, Waltham, MA, USA). Chromatographic separation employed a Waters ACQUITY UHPLC HSS T3 column (1.8 μm, 2.1 mm × 100 mm; Waters Corporation, Milford, MA, USA) thermostated at 40 °C, with a flow rate of 0.2 mL/min. The mobile phase consisted of solvent A (0.1% formic acid in water) and solvent B (0.1% formic acid in acetonitrile). The mass spectrometer was operated with an electrospray ionization (ESI) source, using a capillary voltage of 3800 V in positive-ion mode and −3000 V in negative-ion mode, an ion source temperature of 150 °C, a desolvation gas temperature of 500 °C, and a mass scan range of *m*/*z* 70–1000.

Raw data files (.raw) were processed in Progenesis QI v3.0 (Waters Corporation, USA). Features were filtered by retention time, mass-to-charge ratio, and related parameters; peak areas were corrected using the first QC sample. Peak detection and quantification were performed with a mass tolerance of 5 ppm and a signal intensity deviation threshold of 30%. Molecular formulas were predicted by combining information from molecular ion and fragment ion peaks, and candidate identities were matched against the mzCloud, HMDB, and LIPID MAPS databases. After normalizing the raw quantitative data, compounds with a coefficient of variation (CV) of relative peak area > 30% in QC samples were excluded. Metabolites were thus identified and relatively quantified, and functional annotation was conducted via the KEGG database.

Metabolomics data were further processed using the metaX software (v1.6.0). Principal component analysis (PCA) and partial least squares–discriminant analysis (PLS-DA) were performed to obtain variable importance in projection (VIP) scores. Between-group differences were assessed by *t*-tests, and fold changes (FCs) were calculated. Differential metabolites (DMs) were selected based on VIP > 1.0, FC > 1.2 or FC < 0.833, and *p* < 0.05. After z-score normalization, clustered heatmaps were generated using the R package pheatmap (v1.0.12). Pearson correlation coefficients among DMs were computed with the cor function in R, significance was evaluated using the cor.mtest function (*p* < 0.05), and correlation matrices were visualized with the corrplot package. Functional and pathway analyses of DMs were carried out using the KEGG database, and pathway enrichment bubble plots were generated with the R package ggplot2 (v3.5.0).

### 2.4. Integration Analysis of Transcriptome and Metabolome

To systematically elucidate the gene–metabolite regulatory network associated with differences in growth rate in Dezhou donkeys, we conducted an integrative analysis of transcriptomic and metabolomic data. First, Pearson correlation analysis was used to assess associations between DEGs and DMs, with |r| > 0.8 and *p* < 0.05 set as the criteria for significant correlations, thereby identifying strongly associated DEG–DM pairs. Next, the significantly correlated DEGs and DMs were subjected to KEGG pathway enrichment analysis. Pathway annotation was performed using the KOBAS 3.0 database, and *p* < 0.05 was used as the threshold for significant enrichment. Pathways jointly enriched by both DEGs and DMs were then retained to identify key metabolic pathways and core gene–metabolite interaction relationships governing growth performance in Dezhou donkeys. All analyses were visualized using R (v4.2.1), including correlation heatmaps, KEGG pathway enrichment bubble plots, and interaction network diagrams.

## 3. Results

### 3.1. Transcriptome Sequencing Data Analysis

Using the Illumina NovaSeq 6000 platform with a PE150 strategy, we performed transcriptome sequencing on 12 experimental samples, generating a total of 560,729,784 raw reads, with an average of 42,649,113 reads per sample. After quality filtering with fastp, 541,654,998 clean reads were obtained, with the proportion of clean reads exceeding 94.9% across all samples. The Q30 values for all datasets were greater than 90%, indicating high base-calling accuracy and overall data quality sufficient for downstream bioinformatic analyses (Supplementary Table S1). Clean reads were aligned to the donkey reference genome, yielding a total mapping rate of 91.55% and a unique mapping rate of 89.47% (Supplementary Table S2). These metrics indicate good specificity of alignment to the reference, no evident exogenous contamination, and data stability and reliability meeting the requirements for subsequent analyses.

**Figure 1:**
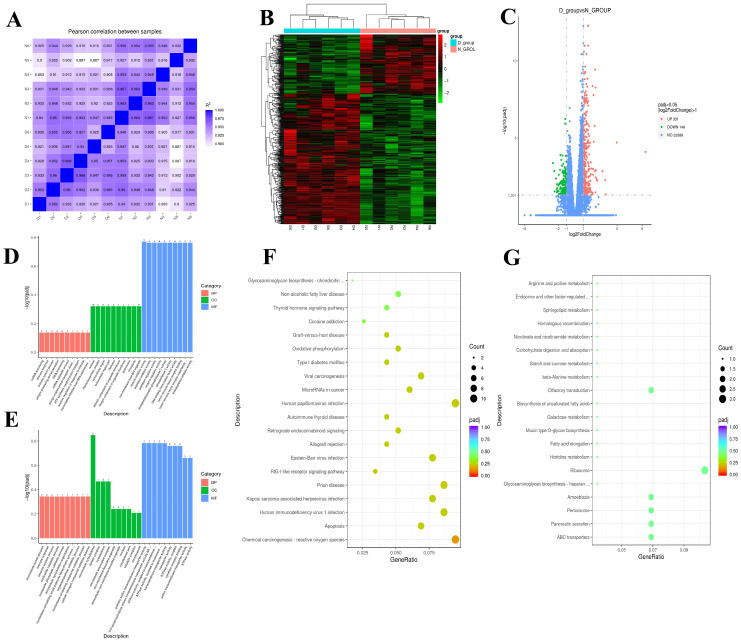
Transcriptomic profiling analysis of plasma samples from Dezhou donkeys with divergent growth rates. (**A**) Pearson correlation heatmap of gene expression among Dezhou donkey samples. (**B**) Hierarchical clustering heatmap of DEGs between D group and N group Dezhou donkeys. (**C**) Volcano plot of DEGs between D group and N group Dezhou donkeys. (**D**) GO enrichment analysis of upregulated DEGs in the D group. (**E**) GO enrichment analysis of upregulated DEGs in the N group. (**F**) KEGG pathway enrichment analysis of upregulated DEGs in the D group. (**G**) KEGG pathway enrichment analysis of upregulated DEGs in the N group.

Correlation analysis of gene expression among samples showed Pearson correlation coefficients greater than 0.88 for all pairwise comparisons, indicating strong biological reproducibility within groups, absence of extreme outliers, and a robust foundation for differential expression analysis (Figure 1A).

Differential expression analysis was performed between group D (Dezhou donkeys with slower growth rate) and group N (Dezhou donkeys with faster growth rate), using |log_2_(Fold Change)| ≥ 1 and adjusted *p*-value (padj) ≤ 0.05 as the screening threshold for significantly differentially expressed genes (DEGs). The results of the clustering heatmap intuitively revealed clear inter-group differentiation in the gene expression profiles of the two groups, along with good consistency of expression patterns among intra-group samples (Figure 1B). A total of 480 significant DEGs were identified between the two groups, among which 331 genes were upregulated in the D group (slow-growing), and 149 genes were downregulated (Figure 1C).

To characterize the biological functional divergence of DEGs between the D and N groups, GO enrichment analyses were performed separately for the upregulated DEGs in each group. The D group’s upregulated DEGs were predominantly enriched in three modules: molecular binding, regulation of enzymatic activity, and immune responses (Figure 1D). Within the molecular binding module, terms such as purine nucleoside binding, GTP binding, and ribonucleoside binding were significantly enriched; within the enzymatic activity regulation module, enzyme inhibitor activity, peptidase inhibitor activity, peptidase regulator activity, and endopeptidase regulator activity were prominent; and within the immune response module, immune response, immune system process, and antigen processing and presentation were significantly enriched. These findings suggest that the slow-growing D group may establish a “metabolic suppression–immune activation” regulatory network by modulating the expression of relevant functional genes; through shifts in energy allocation and suppression of growth-related metabolic pathways, overall growth is ultimately impeded.

In contrast, the N group’s upregulated DEGs were highly enriched in biological processes related to energy metabolism, transmembrane transport, and cytoskeletal organization (Figure 1E). Core enriched terms included primary active transmembrane transporter activity, ATPase activity, coupled to transmembrane movement of substances, and microtubule cytoskeleton. This indicates that the faster-growing N group may enhance the efficiency of energy metabolism and transmembrane transport to more effectively uptake and utilize nutrients required for growth; concurrently, heightened activity of microtubule cytoskeleton-related functions provides structural support for cell proliferation and tissue growth, together constituting a core molecular basis for rapid growth.

Further KEGG pathway enrichment analyses of the upregulated DEGs in each group revealed that the D group was significantly enriched in pathways related to immune responses, apoptosis, and oxidative stress. Key pathways included chemical carcinogenesis—reactive oxygen species, apoptosis, NF-kappa B signaling pathway, and oxidative phosphorylation. Enrichment was also observed in pathogen infection-related pathways such as Human immunodeficiency virus 1 infection and Kaposi sarcoma-associated herpesvirus infection, as well as in disease pathways associated with metabolic dysregulation, including Non-alcoholic fatty liver disease and Diabetic cardiomyopathy (Figure 1F). These results suggest that the D group is subject to combined regulation by oxidative stress and immune activation; sustained immune and stress responses lead to futile energy expenditure accompanied by disturbances in glucose and lipid metabolism, collectively suppressing growth.

The N group’s upregulated DEGs were significantly enriched in pathways associated with substrate transport, nutrient metabolism, and biosynthesis (Figure 1G). Among these, the ABC transporters pathway was significantly enriched, while Pancreatic secretion and Peroxisome approached the threshold for significant enrichment. Pathways related to nutrient metabolism and biosynthesis, such as fatty acid elongation, biosynthesis of unsaturated fatty acids, and Galactose metabolism, also showed enrichment trends. These findings indicate that the N group may increase nutrient uptake efficiency via enhanced activity of the ABC transporters pathway while activating metabolic and biosynthetic pathways for fatty acids and carbohydrates, thereby providing abundant substrates and energy to support rapid growth. The pronounced divergence in KEGG pathway enrichment between the two groups further elucidates, at the signaling pathway level, the core regulatory mechanisms underlying differences in growth rates among Dezhou donkeys.

### 3.2. Metabolomic Profiling of Plasma from Dezhou Donkeys with Different Growth Rates and Analysis of Differential Metabolites

Using liquid chromatography–tandem mass spectrometry, we performed untargeted metabolomics on plasma samples from two groups of Dezhou donkeys exhibiting distinct growth rates: the slower-growing D group and the faster-growing N group (*n* = 6 per group). This approach enabled a systematic characterization of plasma metabolic features associated with divergent growth phenotypes.

**Figure 2:**
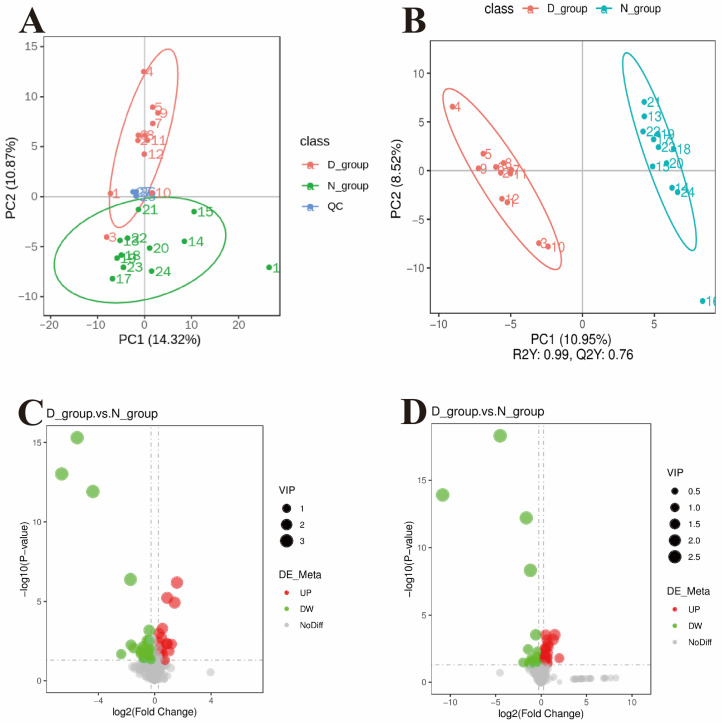
Metabolomic profiling analysis of plasma samples from Dezhou donkeys with divergent growth rates. (**A**) Principal component analysis (PCA) score plot of plasma metabolites. (**B**) Orthogonal partial least squares–discriminant analysis (OPLS-DA) score plot. (**C**) Volcano plot of differential metabolites under positive ionization mode. (**D**) Volcano plot of differential metabolites under negative ionization mode.

Based on relative quantification of metabolites, Pearson correlation coefficients were calculated among quality control (QC) samples. The correlations approached 1 under both positive and negative ionization modes (Supplementary Figure S1A,B), indicating excellent instrument stability throughout the run and reliable data quality. Principal component analysis (PCA) score plots showed tight clustering within groups and a clear separation between the D and N groups, suggesting global differences in plasma metabolic profiles between donkeys with different growth rates (Figure 2A). Orthogonal partial least squares–discriminant analysis (OPLS-DA) further distinguished the two groups, revealing pronounced metabolic divergence with robust model fit and good predictive performance (R2Y = 0.99, Q2 = 0.76). Permutation tests with 200 iterations were conducted to validate the model, and all Q2 values from permutations were lower than the original value, confirming no overfitting, supporting the use of this model for subsequent screening and analysis of differential metabolites (Figure 2B).

In total, 324 metabolites were identified in the positive ion mode and 279 in the negative ion mode. Significantly differential metabolites between groups were screened using variable importance in projection (VIP) > 1.0, fold change (FC) > 1.2 or < 0.833, and *p* < 0.05 as thresholds. In the positive ion mode, 19 metabolites were significantly upregulated and 30 were significantly downregulated in the D group (Figure 2C); in the negative ion mode, 34 metabolites were significantly upregulated and 24 were significantly downregulated in the D group (Figure 2D). Classification annotation of all significant differential metabolites showed that lipids and lipid-like molecules accounted for the largest proportion (44.9%), followed by organic heterocyclic compounds (12.2%) and organic acids and derivatives (8.2%); smaller numbers of metabolites were classified as alkaloids and derivatives, nucleosides and nucleotides, etc., while 24.5% remained unclassified.

Further analysis of group-wise expression characteristics of differential metabolites showed that, in the positive ion mode, the 19 significantly upregulated metabolites in the D group centered on lipids and lipid-derived molecules, including free fatty acids such as cis-vernolic acid and oleic acid, phosphatidylcholines such as PC (18:4/18:4), and lipid oxidation products including 13-HPODE and 17(S)-HETE. Amino acid and nitrogenous derivatives such as glycyl-phenylalanine and trigonelline were also enriched, suggesting that the slower-growing D group exhibits more active lipid oxidative metabolism and enhanced catabolism of specific amino acids. In negative ion mode, among the 34 significantly upregulated metabolites in the D group, lipid-related metabolites again predominated, including lysophospholipids such as LysoPC 14:0, LPC 18:3, and LPE 16:0, as well as lipid oxidation products such as (±)12(13)-DiHOME and (±)9-HpODE. Concurrent enrichment was observed for organic acid derivatives such as 3-indoleacrylic acid and DL-4-hydroxyphenyllactic acid, key tricarboxylic acid cycle intermediates such as alpha-ketoglutaric acid, and inflammation-related lipid mediators such as thromboxane B2 and prostaglandins. These results further corroborate that the D group is characterized by excessive lipid oxidation, activation of inflammatory responses, and reprogramming of energy metabolism.

In contrast, the faster-growing N group had 30 significantly upregulated metabolites in positive ion mode, spanning a broader array of functional classes, including sphingomyelins (e.g., SM [d24:0/12:1]) and monoacylglycerols as structural lipids with functional roles; key intermediates of fatty acid β-oxidation and energy metabolism such as citrulline and decanoylcarnitine; nutrition-related compounds such as vitamin A (retinol); and bioactive signaling molecules with growth-regulatory and antioxidant activities, such as melatonin and andrographolide. In the negative ion mode, the 24 significantly upregulated metabolites in the N group were strongly concentrated in three core functional modules: efficient energy metabolism, biosynthesis of nutrients, and antioxidant defense. Representative metabolites included key products of glycolysis and carbohydrate metabolism (D-(+)-mannose, D-fructose 1,6-bisphosphate), intermediates of the urea cycle and amino acid metabolism (ornithine, citrulline), endogenous antioxidants (L-ascorbic acid, i.e., vitamin C), sulfur-containing amino acid derivatives (gamma-glutamylmethionine, L-cysteine–glutathione disulfide), and precursors of nucleic acid synthesis (cytidine diphosphate [CDP], inosine). These metabolites directly contribute to energy supply, nutrient transformation and utilization, maintenance of antioxidant homeostasis, and biosynthetic processes relevant to cell proliferation, thereby providing a robust metabolic foundation for rapid growth in the N group.

**Figure 3:**
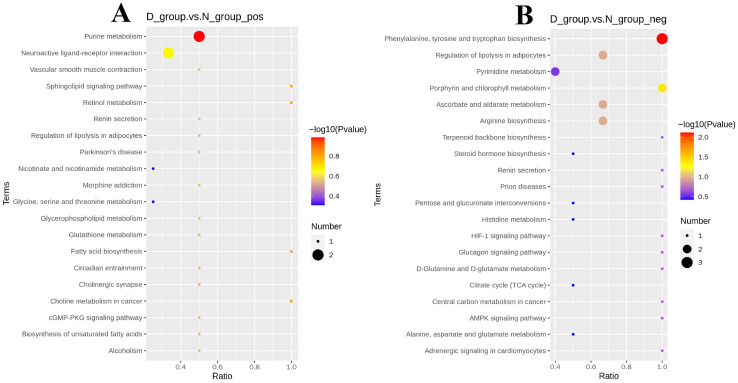
KEGG pathway enrichment analysis of differential metabolites. (**A**) Enrichment bubble plot in positive ion mode, focusing on energy and lipid metabolism pathways. (**B**) Enrichment bubble plot in negative ion mode, emphasizing amino acid metabolism and antioxidant defense pathways.

KEGG pathway enrichment analysis of the significantly differential metabolites revealed marked functional divergence in core regulatory pathways across ionization modes. Differential metabolites identified in the positive ion mode were mainly enriched in pathways related to energy provision and lipid homeostasis, such as purine metabolism, fatty acid biosynthesis, and retinol metabolism (Figure 3A). In negative ion mode, differential metabolites were significantly enriched in pathways central to amino acid metabolism and antioxidant defense, including phenylalanine, tyrosine and tryptophan biosynthesis; porphyrin and chlorophyll metabolism; ascorbate and aldarate metabolism; and arginine biosynthesis (Figure 3B).

Integrative analysis combining group-wise differential metabolite profiles and pathway enrichment results showed that the slower-growing D group exhibits a metabolic network skewed toward excessive lipid oxidation, activation of inflammation-related pathways, and accumulation of catabolic products from specific amino acids. By contrast, the faster-growing N group is characterized by efficient energy utilization, biosynthesis of nutritional substrates, regulation of antioxidant homeostasis, and pro-growth pathways mediated by bioactive signaling molecules. The pronounced divergence in metabolic regulatory networks between groups delineates the core metabolic phenotypes underlying growth-rate differences in Dezhou donkeys. These findings not only provide key metabolomic evidence for elucidating the molecular regulatory mechanisms of growth traits in this breed but also lay an important foundation for subsequent integrated transcriptome and metabolome analyses, screening of molecular biomarkers associated with growth performance, and the development of precision nutrition strategies.

### 3.3. Integrated Analysis of the Transcriptome and Metabolome

**Figure 4:**
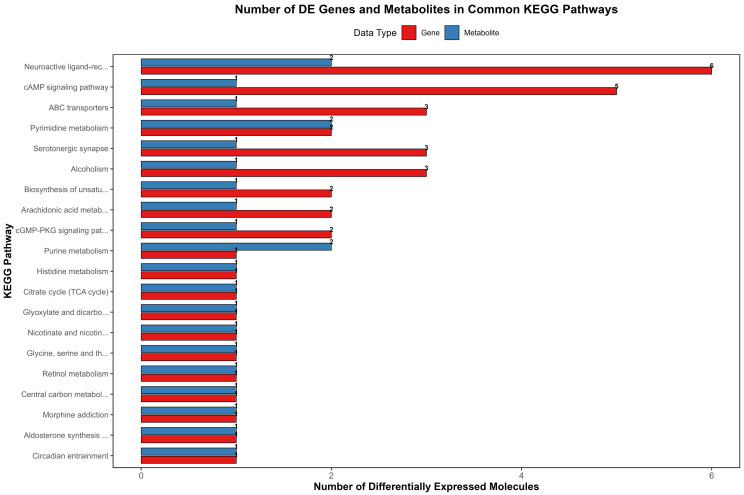
Integrated analysis of DEGs and DAMs in Dezhou donkeys.

To systematically elucidate the metabolic regulatory network underlying growth traits in donkeys, we performed an integrative association analysis of DEGs and DAMs, followed by joint enrichment analysis based on KEGG pathways. The results showed high concordance between pathways enriched in the transcriptomic and metabolomic datasets. The significance of pathways co-enriched by DEGs and DAMs was visualized with a histogram (Figure 4), indicating that the cAMP signaling pathway, arachidonic acid metabolism, and biosynthesis of unsaturated fatty acids are core pathways closely associated with growth rate in donkeys.

Within the arachidonic acid metabolism pathway, the DEGs *LTC4S* and *ALOX15B* act in concert with the DAM thromboxane B2 to regulate processes primarily mediating cell proliferation, inflammatory responses, and lipid metabolism. In the biosynthesis of the unsaturated fatty acids pathway, the DEGs *LOC106827948* and *ELOVL7*, together with the DAM oleic acid, function jointly in maintaining membrane fluidity, hormone synthesis, fat deposition, and growth axis signal transduction, thereby providing a crucial metabolic foundation for growth in donkeys. In the cAMP signaling pathway, five DEGs, namely *JUND*, *BAD*, *LOC123279093*, *CNGB3*, and *ATP2B2*, were significantly co-enriched with the differential metabolite adenosine. As a central signaling hub of the growth axis, this pathway mediates the regulatory effects of growth hormone (GH), IGF-1, and thyroid hormones on growth and development. Additionally, DEGs and DAMs were jointly enriched in the cGMP-PKG signaling pathway, ABC transporters, and neuroactive ligand–receptor interaction, indicating multilevel participation—from signal transduction and substrate transport to neuroendocrine regulation—in the control of growth rate in donkeys.

Further correlation analysis revealed that the key metabolites choline and oleic acid were both significantly positively correlated with *NDUFA11*, *ATP5F1D*, *LOC106828257*, *JUND*, *NDUFS6*, and *SLC7A5* (Supplementary Table S3). In the choline-associated module, *JUND* was enriched in both choline metabolism and the cAMP pathway, making it a critical node linking metabolism with growth signals; the strong association between mitochondrial respiratory chain genes and choline suggests that activation of energy metabolism supports membrane biogenesis and neurotransmitter synthesis; and the positive correlation between *SLC7A5* and choline indicates that enhanced transmembrane transport efficiency is a feature of growth advantage. In the oleic acid-associated module, oleic acid showed strong correlations with *NDUFA11* and *NDUFS6*, implying that energy metabolism and fatty acid synthesis synergistically drive cell proliferation; the association between *JUND* and oleic acid further supports the notion that the cAMP pathway can regulate fatty acid metabolism to promote growth; and the interaction between *SLC7A5* and oleic acid likely contributes to maintaining efficient nutrient utilization.

## 4. Discussion

The Dezhou donkey is a high-quality indigenous livestock breed in China, valued for its combined meat, hide, and medicinal uses. Growth performance is the core trait determining its economic returns. To date, research on growth traits in Dezhou donkeys has focused primarily on phenotypic measurements, breed selection, and dietary nutritional regulation, while the molecular and metabolic regulatory mechanisms underlying divergent growth rates remain insufficiently elucidated. The key functional genes and metabolic targets that govern growth performance also require deeper investigation. In this study, by integrating plasma untargeted metabolomics and transcriptomics, we systematically profiled the metabolic and gene expression signatures associated with differing growth rates, identified core DAMs, DEGs, and key signaling pathways, and delineated a coordinated regulatory network centered on “energy metabolic efficiency–lipid homeostasis–immune/inflammatory regulation–growth signaling.” These findings provide an important theoretical basis and candidate molecular targets for genetic improvement and precision nutritional management of growth traits in Dezhou donkeys, addressing a gap in systematic research in this field.

Lipid metabolism is a central pathway controlling growth and development in livestock. Lipid molecules are not only essential structural components of biological membranes and key vehicles for energy storage, they also act as signaling mediators in inflammation, cell proliferation, and growth regulation. In our metabolomic analysis, lipids and lipid-like molecules accounted for the largest proportion (44.9%) of DAMs between groups with different growth rates [22,23]. The slow-growth group exhibited significant enrichment of free fatty acids, lysophospholipids, lipid peroxidation products, and inflammation-associated lipid mediators, whereas the rapid-growth group showed marked increases in sphingolipids, structural lipids with functional roles, and key intermediates of fatty acid beta-oxidation. This pattern indicates that divergence in lipid metabolic homeostasis is a core metabolic phenotype underlying differences in growth rate in Dezhou donkeys. Prior studies have demonstrated a strong synergy between excessive lipid oxidation and chronic inflammation in livestock, with a positive feedback loop that jointly impedes growth and development. In this study, lipid peroxidation products (such as 13-HPODE and 17(S)-HETE) and pro-inflammatory lipid mediators (thromboxane B2 and prostaglandins) were significantly elevated in the D group and can activate pro-inflammatory signaling pathways including NF-κB [24], thereby inducing persistent low-grade inflammation and immune stress. Notably, 13-HPODE has been shown to directly activate NF-κB, promote the release of pro-inflammatory cytokines, and amplify inflammatory cascades [25]. These findings are highly consistent with transcriptomic results showing significant enrichment of D-group DEGs in NF-κB signaling, immune responses, and apoptosis, directly substantiating the presence of a “lipid oxidation–inflammation activation” positive feedback loop in slow-growing animals. According to the energy allocation trade-off theory of the immune-growth axis in livestock, persistent immune stress in the slow-growing D group diverts nutrients and energy originally allocated to muscle growth and protein synthesis toward immune defense, which leads to wasteful energy expenditure, the suppression of growth-related anabolic processes, and ultimately a reduction in growth rate [26]. The concurrent enrichment of the TCA cycle intermediate alpha-ketoglutaric acid in the D group further suggests a metabolic shift toward this key TCA intermediate, indicative of energy metabolic reprogramming, reinforcing the conclusion that inflammation-driven energy reallocation is a core mechanism restricting growth in these individuals [27]. By contrast, the N group exhibited significant increases in oleic acid and other unsaturated fatty acids that not only maintain membrane fluidity and integrity but also serve as endogenous anti-inflammatory mediators capable of suppressing activation of NF-κB and other pro-inflammatory pathways, thereby reducing inflammatory energy losses and establishing a stable internal milieu conducive to growth [28]. These results align with prior findings in cattle and sheep showing that unsaturated fatty acids can enhance growth performance, and they provide direct metabolomic support for improving Dezhou donkey growth via dietary supplementation with functional unsaturated fatty acids.

Beyond lipid metabolism and inflammatory homeostasis, energy metabolic efficiency and nutrient uptake/utilization capacity constitute another fundamental biological basis for growth potential in livestock. Integrated transcriptomic and metabolomic analyses in this study revealed that the molecular features of rapid-growing N-group animals are concentrated on efficient energy use, transmembrane transport of substrates, and biosynthetic processes, forming a clear functional contrast with the D group [29]. Transcriptomically, DEGs upregulated in the N group were significantly enriched in the ABC transporter pathway, as well as in functional categories such as primary active transmembrane transport and ATPase-coupled transmembrane transporter activity. ABC transporters are a key protein family governing transmembrane transport of nutrients, and their expression directly affects intestinal absorption of amino acids, carbohydrates, and lipids [30]. Multiple studies have shown that activation of ABC transporter pathways is positively associated with growth performance in large livestock species such as cattle and sheep. In this study, activation of ABC transporters functionally aligned with the metabolomic enrichment of amino acid intermediates and carbohydrate metabolites in the N group, suggesting that rapid-growing Dezhou donkeys enhance transmembrane nutrient transport to improve dietary nutrient utilization, thereby providing sufficient substrates for growth and development [31,32]. Moreover, the amino acid transporter gene *SLC7A5* was upregulated in the N group and positively correlated with key metabolites such as choline and oleic acid, further underscoring the pivotal role of transport capacity in growth regulation. The upregulation of *SLC7A5* in the N group facilitates the cellular uptake of leucine, which in turn triggers mTOR-mediated protein synthesis, a central hub for cell proliferation and muscle growth [33,34]. This identifies a key candidate target for elucidating the molecular mechanisms underpinning superior growth in Dezhou donkeys. Mitochondria are the core organelles of energy metabolism, and the efficiency of oxidative phosphorylation directly determines ATP production, a critical factor influencing growth performance. In our study, genes encoding subunits of the mitochondrial respiratory chain, including *NDUFA11* and *NDUFS6* (Complex I) as well as *ATP5F1D* (Complex V), exhibited positive correlations with oleic acid and choline and tended to be upregulated in the N group [35]. The coordinated high expression of these genes and the corresponding elevated metabolite levels suggest more active mitochondrial function and higher energy metabolic efficiency in fast-growing individuals, which guarantees sufficient ATP supply for cell proliferation and tissue growth and forms the energetic basis for their growth advantage. Additionally, the N group exhibited significant enrichment of endogenous antioxidants such as L-ascorbic acid and glutathione derivatives, which can scavenge reactive oxygen species and maintain redox homeostasis, thereby protecting mitochondrial structure and function, further sustaining efficient energy metabolism in synergy with the observed gene expression patterns [36].

Multi-omics integration enables mechanistic dissection of complex economic traits from “gene–metabolite–pathway” perspectives, overcoming the limitations of single-omics analyses. Through integrated enrichment analyses, we found a high degree of concordance between pathways enriched in the transcriptome and metabolome. The cAMP signaling pathway, arachidonic acid metabolism, and biosynthesis of unsaturated fatty acids emerged as core pathways modulating growth rate in Dezhou donkeys. Furthermore, *JUND*-mediated cross-talk between metabolic signaling and growth regulation, together with the synergy between mitochondrial energy metabolism and lipid metabolism, appears to constitute a core molecular module determining growth performance. The cAMP signaling pathway is a central hub along the growth axis, mediating downstream responses to key growth-regulating hormones such as growth hormone (GH) and insulin-like growth factor 1 (IGF-1), thereby controlling cell proliferation, differentiation, and metabolism [37]. In our study, the cAMP pathway was enriched for numerous DEGs and DAMs. The transcription factor gene *JUND* was enriched in both the cAMP signaling and choline metabolism pathways and showed positive correlations with key metabolites (choline and oleic acid), indicating that *JUND* acts as a critical node linking metabolic cues to growth control [38]. As a member of the AP-1 transcription factor family, *JUND* participates in the regulation of cell proliferation, apoptosis, and inflammation. It may interact with key effector proteins within the cAMP pathway to integrate choline metabolic signals with the growth axis and modulate the expression of downstream growth-related genes, thereby amplifying the growth-promoting effects of metabolites. This finding provides a new perspective for understanding the transcriptional regulation of growth traits in Dezhou donkeys. Joint enrichment of the arachidonic acid metabolism and biosynthesis of unsaturated fatty acids pathways further underscores the central role of lipid metabolism in growth regulation [39]. Within arachidonic acid metabolism, DEGs including *LTC4S* and *ALOX15B* were upregulated in the D group, and together with the differential metabolite thromboxane B2, coordinately regulate this pathway, which is not only pivotal in inflammation but also directly involved in lipid metabolism and cell proliferation; its overactivation likely contributes to the inflammatory state and disrupted inflammatory homeostasis observed in slow-growing animals. Within the biosynthesis of unsaturated fatty acids pathway, DEGs including *LOC106827948* and *ELOVL7* act in concert with the differential metabolite oleic acid. *ELOVL7* is a rate-limiting enzyme in the elongation of long-chain unsaturated fatty acids; its elevated expression promotes the synthesis of functional unsaturated fatty acids and aligns with the observed increase in oleic acid in the N group, jointly maintaining membrane homeostasis and mediating growth signaling to support rapid growth. Additionally, DEGs and DAMs were co-enriched in the ABC transporter pathway, cholinergic synapse, and the cGMP–PKG signaling pathway, collectively constructing a multidimensional regulatory network of growth performance from the perspectives of substrate transport, neuroendocrine regulation, and intracellular signal transduction, and systematically revealing molecular metabolic differences underlying divergent growth rates.

Despite systematically elucidating molecular and metabolic mechanisms underlying divergent growth rates in Dezhou donkeys, this study has limitations. First, plasma reflects the organismal metabolic and gene expression profile but cannot capture tissue-specific regulatory mechanisms in key growth-related organs such as muscle, liver, and intestine. Future work integrating multi-omics analyses of target tissues will enable more precise dissection of growth regulatory networks. Second, the biological functions of the core genes and metabolites identified here require further validation. Follow-up studies using cell-based overexpression/RNA interference assays and animal dietary intervention trials are needed to clarify the functions and mechanisms of key candidate targets. Lastly, the growth-associated molecular markers identified in this study should be validated in larger and more diverse Dezhou donkey populations to evaluate their utility and generalizability in genetic breeding programs.

## 5. Conclusions

This study systematically integrates plasma untargeted metabolomics and transcriptomic data to elucidate the core molecular–metabolic mechanisms underlying differences in growth rate in the Dezhou donkey. Slow-growing individuals are characterized by excessive lipid oxidation, disrupted inflammatory homeostasis, and a shift in energy allocation, whereas fast-growing individuals enhance the efficiency of energy metabolism, increase nutrient transport capacity, and maintain redox and inflammatory homeostasis, collectively establishing a molecular–metabolic network conducive to growth and development. The core potential biomarkers identified were *JUND*, *NDUFA11*, *SLC7A5* (genes) and oleic acid and choline (metabolites), which may serve as important targets for genetic improvement and precision nutritional regulation in the Dezhou donkey, providing a key theoretical basis for the efficient and healthy development of the donkey industry. This study not only offers a new perspective on the molecular regulation of growth traits in the Dezhou donkey but also proposes new targets for future breeding and precision nutrition.

## Data Availability

The data presented in this study are available on request from the corresponding author.

## Supplementary Materials

The following supporting information can be downloaded at: https://www.mdpi.com/article/10.3390/ani16081271/s1, Figure S1: Pearson correlation analysis of quality control (QC) samples under different ionization modes. (A) Pearson correlation heatmap among negative ionization mode QC samples. (B) Pearson correlation heatmap among positive ionization mode QC samples; Table S1: Quality control statistics of transcriptomic sequencing data; Table S2: Statistics of reads mapped to the donkey reference genome. Table S3: Correlation analysis results of differential metabolites and differential genes.

## Abbreviations

The following abbreviations are used in this manuscript:

ADG	Average Daily Gain
AMP	Adenosine Monophosphate
ATP	Adenosine Triphosphate
cAMP	Cyclic Adenosine Monophosphate
cGMP	Cyclic Guanosine Monophosphate
CDP	Cytidine Diphosphate
DEGs	Differentially Expressed Genes
DAMs	Differentially Accumulated Metabolites
DMs	Differential Metabolites
ESI	Electrospray Ionization
FC	Fold Change
FPKM	Fragments Per Kilobase of Transcript Per Million Mapped Reads
GH	Growth Hormone
GO	Gene Ontology
GSEA	Gene Set Enrichment Analysis
IGF-1	Insulin-like Growth Factor 1
KEGG	Kyoto Encyclopedia of Genes and Genomes
LC-MS/MS	Liquid Chromatography-Tandem Mass Spectrometry
LysoPC	Lysophosphatidylcholine
LPE	Lysophosphatidylethanolamine
mTOR	Mammalian Target of Rapamycin
NDUFA11	NADH:Ubiquinone Oxidoreductase Subunit A11
NDUFS6	NADH:Ubiquinone Oxidoreductase Subunit S6
NF-κB	Nuclear Factor-kappa B
OPLS-DA	Orthogonal Partial Least Squares–Discriminant Analysis
PCA	Principal Component Analysis
PC	Phosphatidylcholine
PE	Paired-End
PKG	Protein Kinase G
PLS-DA	Partial Least Squares–Discriminant Analysis
QC	Quality Control
SLC7A5	Solute Carrier Family 7 Member 5
SM	Sphingomyelin
TCA	Tricarboxylic Acid
VIP	Variable Importance in Projection
