# Supplementary figures and images for: Integrated Transcriptomic and Metabolomic Analysis Deciphers the Molecular and Metabolic Mechanisms Underlying Growth Rate Divergence in Dezhou Donkeys
Source: Animals (Basel). 2026 Apr 21;16(8):1271. doi: 10.3390/ani16081271 (PMC13113997; doi:10.3390/ani16081271)

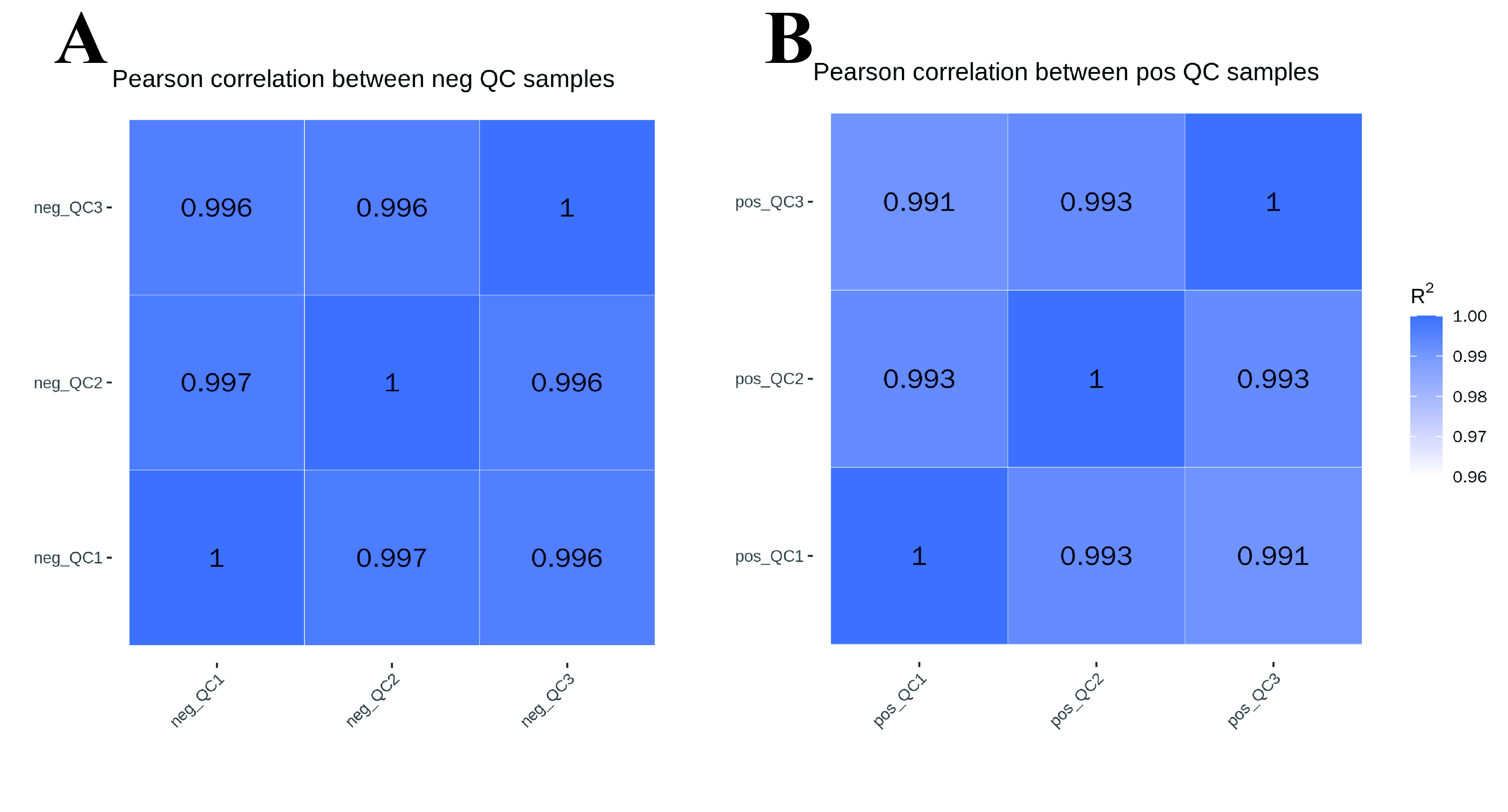

Supplement: Supplementary file 1 [file animals-16-01271-s001.zip › Supplementary Figure S1.jpg]
